# Programmable broad-spectrum resistance to bacterial blight using targeted insertion in rice

**DOI:** 10.1038/s41421-024-00714-8

**Published:** 2024-10-08

**Authors:** Xuening Zhang, Minglei Song, Yingying Wang, Qi yao, Rundong Shen, Yifu Tian, Yuming Lu, Jian-Kang Zhu

**Affiliations:** 1 CAS Center for Excellence in Molecular Plant Sciences, University of Chinese Academy of Sciences, Shanghai, China; 2 Tea Research Institute, Chinese Academy of Agricultural Sciences, Hangzhou, Zhejiang, China; 3 Institute of Advanced Biotechnology, Southern University of Science and Technology, Shenzhen, China; 4 School of Agriculture and Biology, Shanghai Jiao Tong University, Shanghai, China

**Keywords:** Plant molecular biology, Pattern recognition receptors in plants

Dear Editor,

Rice bacterial blight, caused by *Xanthomonas oryzae pv. oryzae (Xoo)*, results in over 50% yield loss in Asia and Africa^1^. *Xoo* injects transcription activator-like effectors (TALEs) into plant cells to initiate gene transcription by targeting effector binding elements (EBEs) via repeat variable di-residues (RVDs)^2^. CRISPR/Cas9-mediated gene editing allows precise deletion and insertion of genomic sequence in rice^3^. While knocking out EBEs in susceptibility genes can increase bacterial blight resistance in rice^4^, the knockout may cause yield penalties since some susceptibility genes are important for rice growth and development. Incorporating EBE sequences into executor (E) genes is another strategy for broad-spectrum resistance by enabling rice to trap TALE-containing strains. For instance, rice lines incorporating three EBEs recognized by AvrXa23, AvrXa7, and TalC respectively exhibited resistance to multiple *Xoo* strains^5–7^. However, the insertion of a limited number of EBEs currently cannot cope with the vast *Xoo* population diversity.

In this work, we employed CRISPR/Cas9 to knock in multiple EBEs into rice E gene promoters, generating programmed broad-spectrum resistance against diverse *Xoo* strains. We show that our strategy of combining EBE deployment with pathogen monitoring through genome sequencing could enable countering evolving *Xoo* populations in rice (Fig. 1a).

**Fig. 1:**
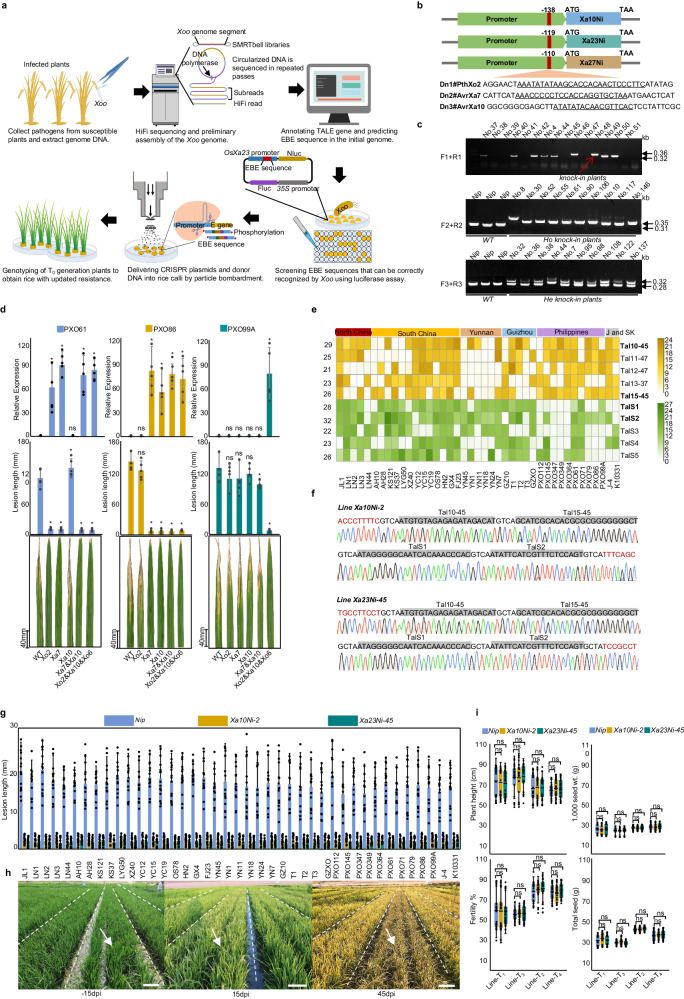
Programmed resistance to bacterial blight in rice using EBE knockin strategy. **a** Schematic overview of programmed resistance to bacterial blight in rice using targeted insertion. **b** Target sites and donor DNA sequences for *Xa10Ni*, *Xa23Ni*, and *Xa27Ni*. Red lines indicate insertion sites, located 138 bp, 110 bp, and 119 bp upstream of the start codons of the corresponding E genes. The length of single EBE DNA donors was 40 bp. **c** PCR amplification of target sites to determine the presence of insertions in T_0_ plants. The larger-band or double-band samples are selected for sequencing (top). Single bands indicate homozygous insertion (middle) and double bands indicate heterozygous insertion (bottom). Primers F1/R1, F2/R2 and F3/R3 are flanking the insertion site of *Xa27Ni*, *Xa23Ni*, and *Xa10Ni* respectively. *Nip* indicates the wild type of *Nipponbare*. Each number represents a T_0_ plant. **d** Activation of resistance in five EBE knockin lines upon inoculation with PXO61, PXO86, and PXO99A. Error bars indicate SD (*n* = 5). *P-*values were calculated by unpaired *t*-test; ns, not significant; *P < 0.05. **e** Relative luciferase activity of calli carrying the EBE reporter after inoculated with 41 *Xoo* strains. The yellow block indicates the calli with a reporter gene driven by promoters containing the EBE recognized by Tal10-45, Tal11-47, Tal12-47, Tal13-37, or Tal15-45 (Identified in *Xoo* isolates mainly from the Philippines). The green block indicates the calli with a reporter gene driven by promoters containing the EBE recognized by TalS1, TalS2 TalS3, TalS4, or TalS5 (Identified in *Xoo* isolates from China). **f** Sanger sequencing confirmation of EBE insertions in T_0_ plants of *Xa10Ni-2* and *Xa23Ni-45* lines. **g** Lesion lengths 15 days after inoculation of *Xa10Ni-2* and *Xa23Ni-45* T_4_ lines with 41 *Xoo* strains., Error bars indicate SD (*n* = 15). **h** Field phenotypes of *Xa10Ni-2* and *Xa23Ni-45 T*_*4*_ lines at 15 days before inoculation, 15 days after inoculation, and 45 days after inoculation. White arrows indicate unedited controls. Each line was planted in 1.2 m × 30 m plots and inoculated in blocks with each *Xoo* strain over 0.75 m sections containing ~50 plants, scale bar, 12 cm. **i** Agronomic traits of *Xa10Ni-2* and *Xa23Ni-45* T_1_‒T_4_ lines. T_1_ and T_3_ generations were grown in Hainan (with relatively low fertilities caused by high temperature on the island), while T_2_ and T_4_ were grown in Shanghai; error bars indicate SD (*n* = 15), with *P*-values from unpaired *t*-test at the significance level of 0.05; ns, not significant.

To assess the potential of engineering rice endogenous E genes for achieving resistance against *Xoo*, we designed seven distinct donor DNA fragments (Dn#1~Dn#7), each containing single or multiple unique EBEs (Supplementary Table S1), for targeted knockin. Corresponding CRISPR/Cas9-sgRNA vectors were designed for targeting the promoters of three E genes (*Xa10Ni*, *Xa23Ni*, and *Xa27Ni*, Fig. 1b), and each was co-transformed with different donor DNA into rice calli via bombardment. Genotyping of 2042 T_0_ plants using donor-specific primers showed that effective knockin was achieved for all seven DNA donors at all three target genes, with efficiencies ranging from 5.3% to 23.2% (Fig. 1c; Supplementary Fig. S1 and Table S1).

To evaluate the knockin-induced immunity against specific *Xoo* strains, we self-pollinated the T_0_ plants harboring the desired Dn1#E1Xo2 insertions and inoculated their T_1_ progenies with *Xoo* strain PXO61 which contains the TALEs recognizing EBEs Xo2 and Xa7. qPCR results revealed that all three E genes with Dn1#E1Xo2 insertions were upregulated upon inoculation in T_1_ plants. *Xa10Ni*^*Xo2*^ and *Xa23Ni*^*Xo2*^ lines exhibited resistance to PXO61, while *Xa27Ni*^*Xo2*^ remained susceptible (Supplementary Fig. S2), indicating that *Xa27Ni* from *Nipponbare* is a nonfunctional *Xa27* variant likely due to sequence divergence from the original *Xa27* gene.

Subsequently, the T_1_ progenies of another four knockin lines were inoculated with distinct *Xoo* strains to evaluate the impact of EBE diversity on *Xoo* resistance (Fig. 1d; Supplementary Fig. S3). As expected, *Xa23Ni*^*Xa7*^ and *Xa23Ni*^*Xa10*^ lines exhibited resistance against PXO86 that harbors the TALEs recognizing EBEs Xa7 and Xa10. Except for the *Xa23Ni*^*Xo2&Xa10&Xo6*^ line that exhibited resistance to PXO99A carrying TALEs recognizing EBE Xo6, the other four knockin lines are susceptible to this strain (Fig. 1d). This suggests the programmable nature of EBE-mediated immunity, which allows flexible EBE design based on prevailing *Xoo* TALE repertoires.

We then explored whether the tandem insertion of multiple EBEs could broaden resistance to *Xoo* in rice. Two knockin lines with four-EBE insertions, *Xa10Ni*^*E4*–*11*^ (containing EBE Xo7, Xa10, Xo6, Xa27) and *Xa10Ni*^*E4-14*^ (containing EBE Xo1, Xo2, Xa7, TalC), were selected and inoculated with 41 diverse *Xoo* isolates from East Asia that were available to our laboratory (Supplementary Fig. S4 and Table S2). *Xa10Ni*^*E4-11*^ lines exhibited resistance against 21 strains, and *Xa10Ni*^*E4-14*^ lines resisted 26 strains (Supplementary Fig. S5). Subsequent agronomic trait assessments showed no adverse impacts for either multi-EBE line (Supplementary Fig. S6). Collectively, these results demonstrated that although targeted EBE insertion is an effective strategy for programmable resistance against *Xoo* in rice, additional EBEs are needed for engineering rice resistant to all 41 *Xoo* strains.

To validate whether a rational design incorporating sequencing data could further extend the availability of EBEs for knockin, we analyzed the RVD sequences of TALEs from 84 *Xoo* strains with published genomes. We observed correlations between TALE types and geographical origins (Supplementary Fig. S7). TalC and TalF were found to predominate in African strains, while executor-targeting TALEs were absent among African isolates but were widespread in Asian strains. SWEET-targeting TALEs displayed immense diversity among Asian isolates. According to the RVD sequence conservation, we identified eight novel TALEs prevalent in strains from proximate regions (Supplementary Fig. S8 and Table S3). For example, Tal4-27 and Tal17-37 were highly conserved among African isolates. Tal15-45 was conserved in Philippine strains, while Tal11-47 was conserved in isolates from Southeast Asia, Japan and Korea, but not from China or India, likely reflecting diversified rice genetic backgrounds across these regions. Such geographical patterns of TALE populations may reflect regional evolution and adaptation of *Xoo*^8^.

To determine whether these predicted TALEs can target the EBE designed according to RVD‒DNA pairing rules, we developed the modular EBE assembly into a Dual-Luc reporter system (Supplementary Fig. S9a) and introduced it into rice calli. Activation of the reporter was detected in EBE-carrying calli upon infection with the respective *Xoo* strains (Supplementary Fig. S9b, c). A twenty-fold luciferase activity increase was observed after 3 days (Supplementary Fig. S9d). Therefore, five putative conserved EBEs identified from Asian *Xoo* were tested against the 41 *Xoo* strains (Fig. 1e). The assays showed that the EBEs were responsive to *Xoo* strains from some regions only, reflecting the strong regional evolution of Chinese *Xoo*. Thus, these initial EBEs failed to confer a response against all 41 strains.

To identify EBEs responsive to all 41 *Xoo* strains, PacBio HiFi sequencing was employed to sequence 30 diverse Chinese unsequenced *Xoo* isolates^8^, yielding 22 G reads. The sequencing achieved 99.9% accuracy at 10‒25 kb reads spanning the 2‒4 kb TALE genes. The final assemblies were obtained by filtering out contigs with short sequence lengths. We extracted TALE genes from each draft genome and analyzed their RVD sequences. The distribution of known TALEs such as PthXo1, PthXo2, TalC, PthXo6, PthXo7, AvrXa10, and AvrXa27 in the 41 strains fully explains the resistance and susceptibility phenotypes observed in lines *Xa10*^*E4-11*^ and *Xa10*^*E4-14*^ (Supplementary Fig. S10 and Table S6). We selected the top five most frequently occurring novel TALEs, designated as TalS1‒TalS5, and incorporated their corresponding EBEs into the aforementioned reporter system (Supplementary Fig. S11a, b). The results indicate that these reporters exhibit a higher rate of activation when exposed to Chinese strains, as opposed to strains from other regions (Fig. 1e). EBEs for four TALEs, TalS1, TalS2, TaI15-45 and Tal10-45, were chosen for generating the knockin lines because they can confer a response to all 41 strains. Accordingly, they were combined and inserted into *Xa10Ni* and *Xa23Ni* promoters for generating homozygous *Xa10Ni-2* and *Xa23Ni-45* knockin lines (Fig. 1f). Cas9-free T4 lines exhibited high resistance against all 41 strains in field tests (Fig. 1g, h), indicating that sequencing-guided EBE deployment is effective for precision elevation of broad-spectrum resistance by trapping conserved *Xoo* TALEs. Continual population sequencing to monitor *Xoo* variation could enable ongoing updates to EBE deployment, addressing the issue that rice show resistance breakdown by emerging strains.

Leakage expression of transgenic E genes could be lethal to plants. In contrast, EBE knockin is considered to show no deleterious effects, given that its activation relies on TALE. Field experiments confirmed the absence of toxicity in lines harboring knockin of 1‒4 concurrent EBEs at *Xa10Ni* and *Xa23Ni* (Supplementary Fig. S12 and Table S7) Continued monitoring of resistance and field phenotypes across T_1_‒T_4_ generations of *Xa10Ni-2* and *Xa23Ni-45* lines further validated the heritability and stable efficacy of programmable immunity (Supplementary Fig. S13), with no lethal plants observed in the field. Moreover, targeted next-generation sequencing (NGS) results of putative off-target sites showed no detectable off-target editing in these two EBE-inserted lines (Supplementary Table S8) The knockin lines do not show substantial differences as compared with wild-type plants in agronomic traits, including grain weight and yield. (Fig. 1i; Supplementary Fig. S14a, b). Crossing the T-DNA-free knockin lines into elite rice varieties will facilitate rapid resistance deployment in the future.

In this study, we investigated the feasibility of creating a programmable and heritable rice immune system by engineering “promoter trap” EBEs to induce E genes. We employed PacBio HiFi sequencing to identify novel conserved TALEs and successfully generated EBE-inserted lines that show resistance against diverse *Xoo* populations with sequencing-guided EBE deployment. However, the resistant lines generated by bombardment-mediated transformation may harbor multiple copies of the exogenous sequence. Crossing these lines with elite varieties and subsequent backcrosses will be necessary to remove extra copies of the exogenous sequence. In summary, our programmed immunity system, combined with sequencing-guided EBE deployment offers a promising strategy to counter resistance breakdown in rice varieties.

## Supplementary information

Supplementary information

## Data Availability

All data generated or analyzed during this study are included in this manuscript and its supplementary information files. The plasmids used in this study will be available at Addgene, and the materials are available from the corresponding author upon request. Sequence data for this study have been submitted to the NCBI under BioProject accession PRJNA1090237.
