# Supplementary material for: Programmable broad-spectrum resistance to bacterial blight using targeted insertion in rice
Source: Cell Discov. 2024 Oct 8;10:100. doi: 10.1038/s41421-024-00714-8 (PMC11458567; doi:10.1038/s41421-024-00714-8)
Supplement: Supplementary file 1 — Supplementary information [file 41421_2024_714_MOESM1_ESM.pdf]

**Supplementary Information**

# Programmable broad-spectrum resistance to bacterial blight using targeted insertion in rice

Xuening Zhang[1,2,5], Minglei Song[1,3,5], Yingying Wang[1], Qi yao[1], Rundong Shen[1], Yifu Tian[1*], Yuming Lu[4*], Jian-Kang Zhu[3*]

[1]CAS Center for Excellence in Molecular Plant Sciences, University of Chinese Academy of Sciences

[2]Tea Research Institute, Chinese Academy of Agricultural Sciences

[3]Institute of Advanced Biotechnology, Southern University of Science and Technology

[4]School of Agriculture and Biology, Shanghai Jiao Tong University

[5]These authors contributed equally.

Corresponding author: zhujk@sustech.edu.cn; luymin@sjtu.edu.cn; tianyifu@caas.cn.

## Supplementary Materials and Methods

**Plant and *Xoo* strain cultivation**. The rice variety used in this study was Nipponbare (*Oryza sativa L. japonica cv. Nipponbare*). Rice seedlings were grown in growth chambers at 30°C for 12 hr and 26°C for 12 hr until reaching approximately 15 cm in height. The seedlings were then transplanted to Songjiang Farm (average temperature 22-34°C, relative humidity 59%) or Lingshui Farm (average temperature 20-29°C, relative humidity 73%) for further growth until maturity. *Xoo* strains were cultured on PSA medium (10 g/L tryptone, 10 g/L sucrose, and 1 g/L glutamic acid) at 28°C.

**Dual-luciferase assay in rice protoplasts.** Ten-day-old rice seedlings were used for protoplast isolation (grown on 1/2 MS medium with a 16-hr light/8-hr dark cycle). The extraction of protoplasts followed previous methods[1], where tender stems of rice were cut into fine strips and immersed in an enzyme solution (1.5% Cellulase R-10, 0.75% Macerozyme R-10, 0.8 M mannitol, 10 mM MES at pH 5.7, 10 mM $CaCl_2$). The strips were then vacuum infiltrated at -0.04 mpa for 5 minutes and oscillated under dark conditions at 60-80 rpm for 4-5 hours. The enzymatic hydrolysate was filtered into round-bottom tubes using 40-μm filters. The protoplasts were collected by centrifugation at 1500 rpm for 3 minutes and then resuspended using W5 solution (154 mM NaCl, 125 mM $CaCl_2$, 5 mM KCl, and 2 mM MES at pH 5.7). After centrifugation and collection again, the protoplasts were resuspended in MMG solution (0.4 M mannitol, 15 mM $MgCl_2$, and 4 mM MES at pH 5.7). The protoplasts were kept on ice for 30 minutes, then 5 μg plasmid DNA was mixed with 100 μl protoplasts in 220 μl of 40% PEG solution (40% (w/v) PEG 4000, 0.2 M mannitol and 0.1 M $CaCl_2$). The mixture was incubated in the dark at 28°C for 15 minutes, after which the reaction was terminated with 500 μl W5 solution. The protoplasts were centrifuged and then resuspended in 200 μl of W5 solution, then cultured under dark conditions at 28°C for 48 hours. After incubation, protoplasts were lysed to measure Nluc/Fluc activity using the Dual-Luciferase Reporter Assay System (Promega).

**Knock-in rice construction**. The CRISPR-Cas9 vector construction, donor preparation, and particle bombardment transformation were performed as described previously[2]. Briefly, sgRNAs targeting knock-in regions were constructed into the pCBSG032 vector. Insertion sequences were phosphorylated at the 5’ ends, thiol-modified, and annealed in 50 mM NaCl and 1 mM EDTA to form

dsODNs. The CRISPR-Cas9 plasmid and donor DNA were mixed (0.1 pmol plasmid + 10 pmol donor) and added to 50 μL gold particle solution (60 mg/mL). This was used to transform 1-month-old Nipponbare embryogenic calli via the PDS1000/He system (Bio-Rad). Positive knock-in plantlets were obtained from hygromycin-resistant calli. Genomic DNA of $T_0$ plants was extracted by the CTAB method. Target regions were PCR amplified using flanking primers to detect insertions. Sanger sequencing identified plantlets with seamless insertions, which were propagated to obtain $T_1$-$T_4$ generations. Homozygous knock-in lines without Cas9 were selected for experiments. All primers used in this study are listed in Supplementary Table 3.

**Disease assays.** The leaf-clipping method inoculated booting stage rice. *Xoo* strains from -80°C were streaked onto 15 mg/L cephalexin PSA plates. Single colonies were propagated and resuspended in dd$H_2O$ to OD600 0.5. Scissors dipped in bacteria clipped the top 2 cm of leaves at an angle. Inoculated leaves were collected 24 hours later and frozen. Lesion lengths were measured 14 days post-inoculation. 5-10 leaves on 15-30 plants per *Xoo* strain were inoculated.

***Xoo*-EBE activation assay in transgenic rice callus.** Rice seeds were sterilized with 75% ethanol and 5% sodium hypochlorite before sowing on NB medium (NB powder 4.2 g, Sucrose 30 g, 2,4-D 200 μl, Phytagel 3.5 g, dissolved in 1 L water and sterilized). After 2 weeks of cultivation, intact calli were selected and immersed in an infection solution containing *Agrobacterium tumefaciens* with reporter gene plasmids for 5 minutes. The calli were then transferred to moist filter paper and incubated at 21°C for 2 days. Subsequently, the callus tissues were washed in 400 mg/L ampicillin solution and transferred to NB medium containing hygromycin until new callus particles formed. 10 g of transgenic callus tissue was taken for the *Xoo*-EBE activation experiment. The calli were soaked in an *Xoo* suspension for 1 minute, then transferred to moist filter paper at 21°C for 3 days. After lysis, Nluc/Fluc activity was measured using the Dual-Luciferase Reporter Assay System (Promega).

**RNA extraction and RT-qPCR.** Total RNA was extracted using the RNAprePlant kit (TANGEN). cDNA was reverse transcribed, and qPCR was performed using the CFX96 (Bio-Rad). *OsActin1* (*Os03g071810*) was the internal reference with three technical replicates per five plant samples.

**Rice agronomic trait measurements.** Plant height, panicle number, panicle length, and other phenotypes were measured at maturity. Thousand-grain weight was determined, and filling percentage was calculated as unfilled grains/total grains, along with yield per plant. Thirty plants per line were systematically measured.

***Xoo*** **HiFi sequencing, assembly, and TALE identification.** *Xoo* genomic DNA was extracted using the Bacterial gDNA Purification Kit (Vazyme) per the manufacturer's protocol. High-quality DNA (OD260/280 = 1.8-2.0, ≥10 μg) was purified, end-repaired, and ligated with SMRT bell adapters (Pacific Biosciences). The library was purified using 0.45x Agencourt AMPure XP beads (Beckman Coulter). A ~10 kb insert library was sequenced on one SMRT cell via CCS. Reads were processed using SMRT Analysis v2.3.0. Clean short and long reads were co-assembled into contigs with HiFiasm[3]. The assembled genomes were aligned utilizing the genome of PXO99A as a reference. From this alignment, SNPs were extracted to construct maximum likelihood (ML) phylogenies with RAxML.[4] The resulting phylogenetic trees were then visualized and refined using iTOL[5.] AnnoTALE was used to annotate TALEs from contigs[6].

**Copy number and off-target Estimation.** The copy number estimation was described previously[7]. Briefly, the single copy of the rice gene *OsSPS1(LOC_Os01g69030)* was used as the internal reference. The 1900bp fragments of SPS1 and Cas9 were amplified and constructed into pCE2 TA/ blunt zero vector respectively. Linear carrier with gradient dilution was used for quantitative RT-PCR and standard curve production. After confirming the amplification efficiency of Cas9 and SPS1 is similar (0.94 and 0.98, respectively), the copy numbers of EBE, Cas9, and Hyg were determined by comparing the quantified transcripts with those of the endogenous SPS1 gene in terms of the standard curves. Each rice DNA sample was tested three times. The CRISPR-GE[8] （http://skl.scau.edu.cn/offtarget/） was utilized to predict the potential off-target sites. The target region was amplified from genomic DNA with site-specific primers in the first round of PCR. In the next round of PCR, both forward and reverse barcodes were added to the ends of the PCR products using barcode primers. Amplicons were purified using a Monarch PCR & DNA Cleanup Kit (New England Biolabs). Equal amounts of PCR product were sent for NGS using the Hi-TOM platform[9]. Mutations were detected using HiTOM analysis.

**Statistical analysis.** Data were analyzed by one-way ANOVA in ORIGIN, and charts were generated. Tukey's test determined significant differences ($P<0.05$). Significance levels and sample sizes (n) are in the figure legend.

**Supplementary Figure**

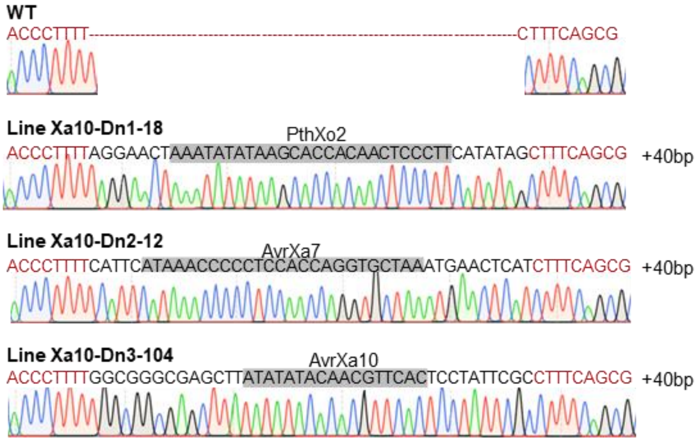


**Supplementary Fig S1. Representative Sanger sequencing chromatograms of EBE-inserted lines.** Inserted EBEs were marked with shadow; non-shaded regions are promoter sequences.

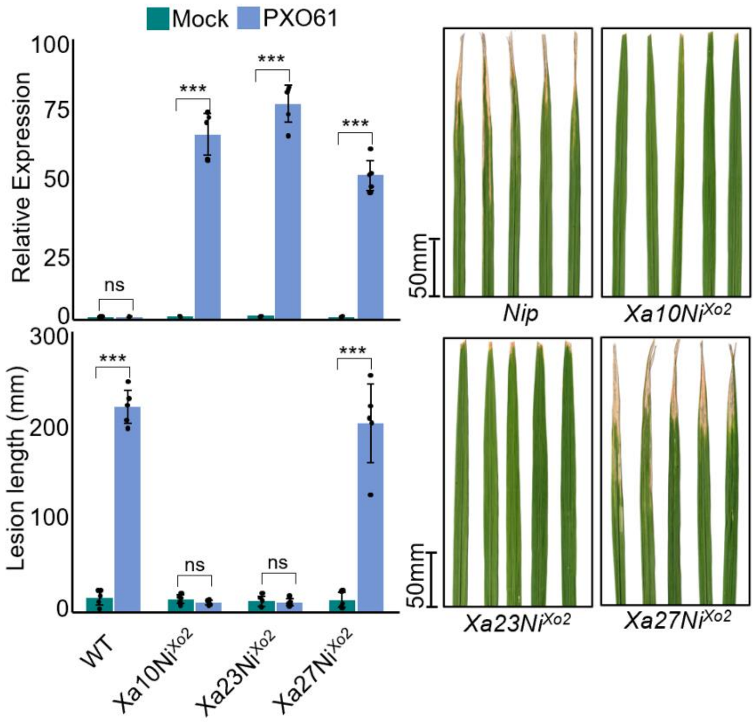


**Supplementary Fig S2. Resistance testing of *Xa10Ni*, *Xa23Ni*, and *Xa27Ni* EBE Xo2 knockin lines.** E gene expression 24 h post-inoculation and lesion lengths 14 days post-inoculation were measured; Error bars indicate SD (n=5) and *p*-values are from *t*-tests at significance level of 0.05.

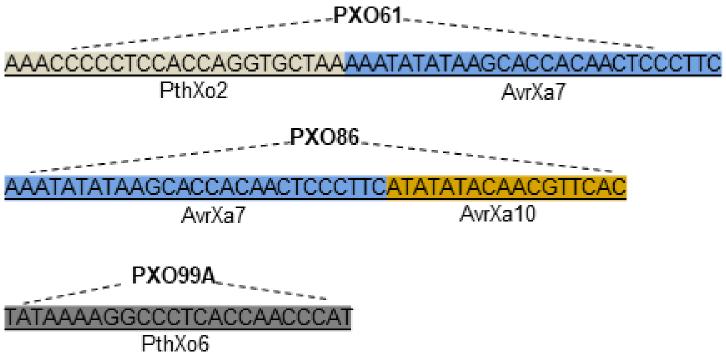


**Supplementary Fig S3. TALEs and their targeted effector binding element of rice in PXO61, PXO86, and PXO99A.**

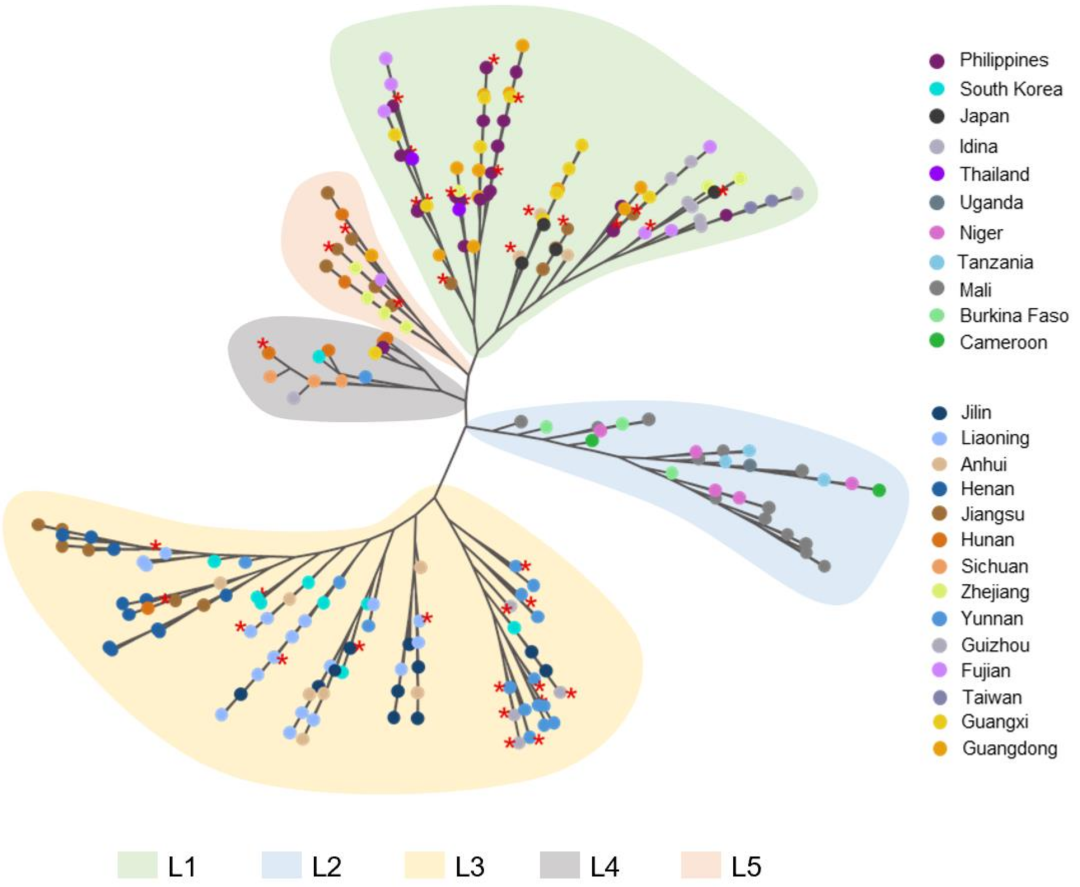


**Supplementary Fig S4. Population genetic analysis of 198 Xoo strains from 25 regions.**

The maximum likelihood tree is based on the core SNPs of the Xoo genome sequences. Colored dots indicate Xoo strains of the corresponding isolation. The five lineages(L1-L5) were decorated with different colors. Strains used in this study were marked with asterisks.

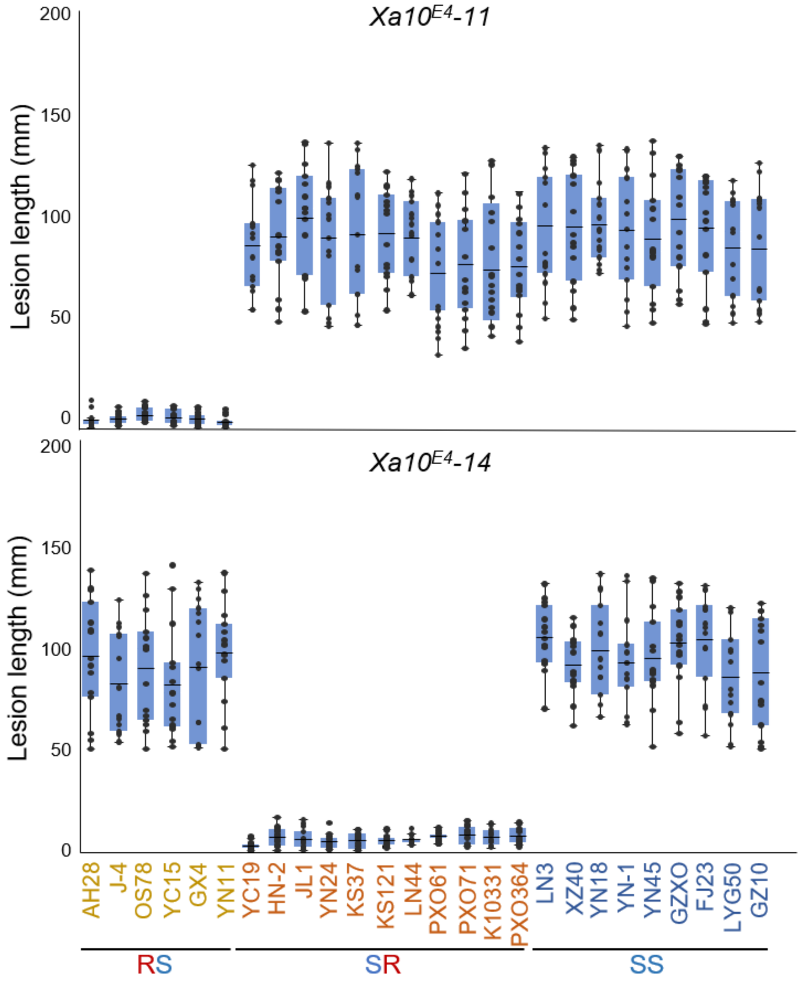


**Supplementary Fig S5. Resistance testing of *Xa10Ni*$^{E4}$*-11* and *Xa10Ni*$^{E4}$*-14* lines inoculated with 41 *Xoo* strains.** *Xa10Ni*$^{E4}$*-11* contains EBEs for Xo7, Xa10, Xo6, Xa27, and *Xa10Ni*$^{E4}$*-14* contains EBEs for Xo1, Xo2, Xa7, TalC. Both showed resistance to 15 strains (data not shown), differential resistance to 17 strains, and susceptibility to 9 strains (n=15).

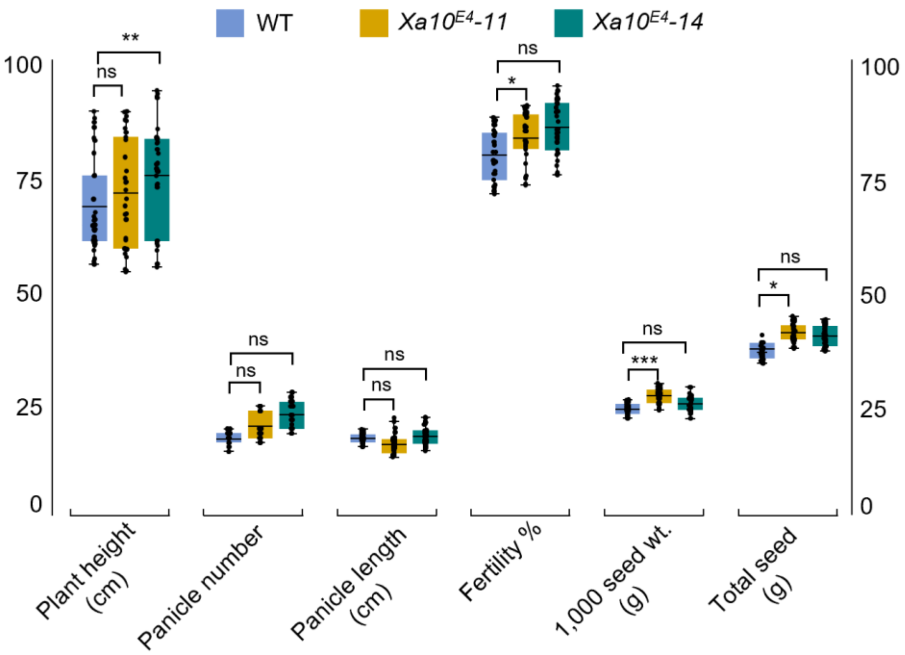


**Supplementary Fig S6. Agronomic traits of *$Xa10Ni^{E4}$-11* and *$Xa10Ni^{E4}$-14* lines.** Plant height, height from ground to top leaf. Panicle length, length from panicle tip to last rachis internode. Fertility, ratio of filled to total seeds. Total seed, total seed weight per plant, error bars indicate SD (n=30), *p*-values from *t*-tests at a significance level of 0.05.

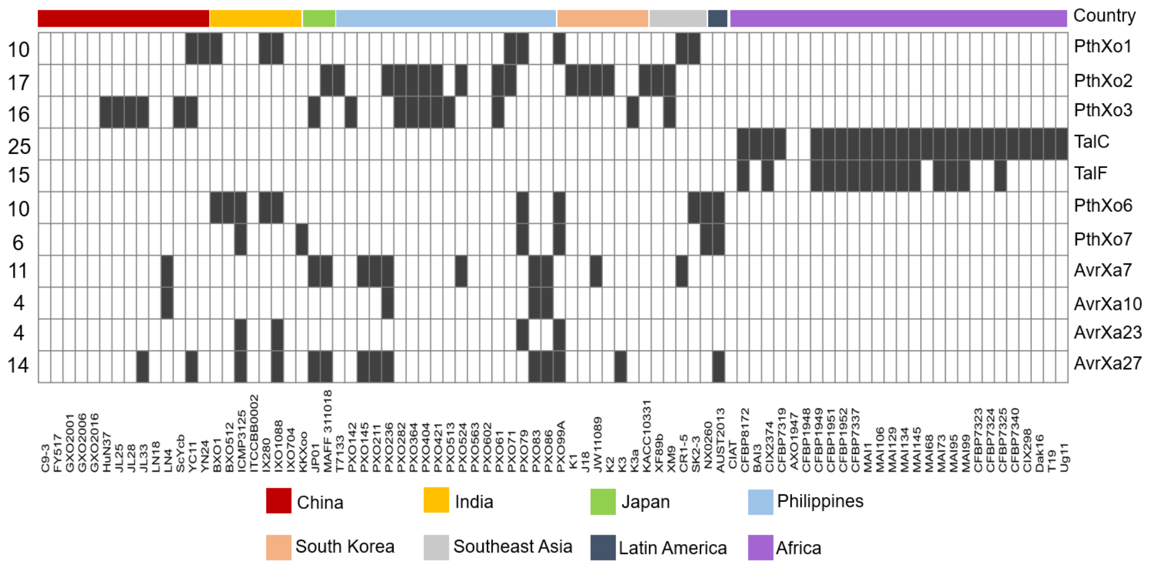


**Supplementary Fig S7. Distribution of TALEs targeting susceptible genes and E genes across 84 *Xoo* strains.** Horizontal axis shows *Xoo* strains. Vertical axis shows TALE genes. Black shading indicates strains containing the corresponding TALE. Colored bars indicate countries where the strains were isolated.

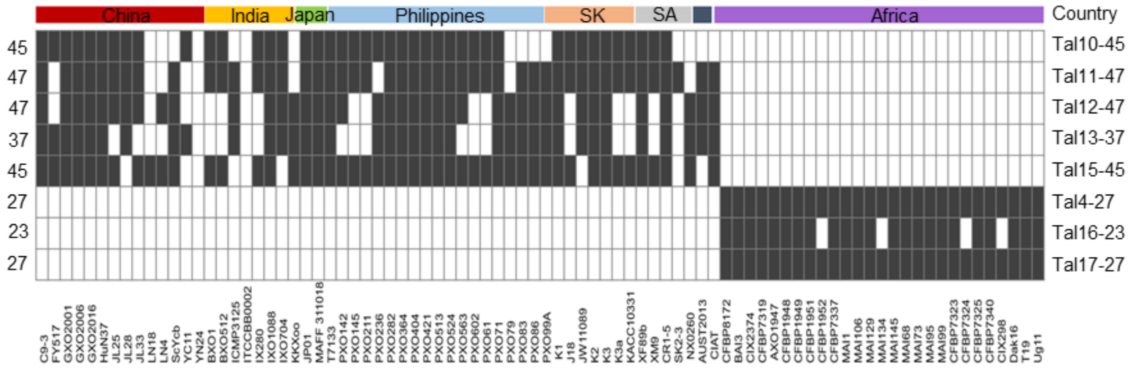


**Supplementary Fig S8 Distribution of 8 novel TALEs across 84 fully sequenced *Xoo* strains by region.** Colored bars indicate the regions where the strains were isolated, SK indicates South Korea, and SA indicates Southeast Asia. Dark shading indicates strains containing the corresponding TALE.

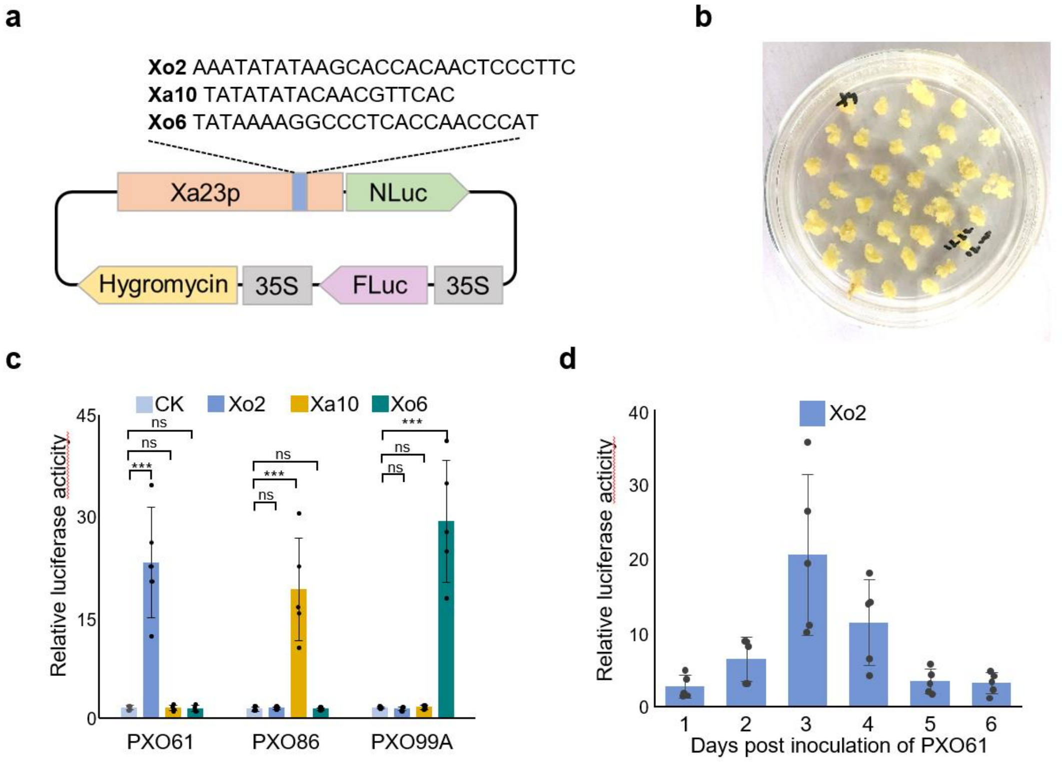


**Supplementary Fig S9. Dual luciferase reporter assay in rice calli. (a)** Schematic of Dual-Luc vector for testing *Xoo* activation of EBEs in transgenic rice callus. **(b)** Rice callus carrying Dual-Luc reporter containing EBEs. **(c)** Relative luciferase activity in callus with EBEs for Xo2, Xa10, and Xo6 after inoculation with PXO61, PXO86, and PXO99A. Control was an empty reporter without EBEs, error bars indicate SD (n=5), *p*-values from *t*-tests at a significance level of 0.05. **(d)** Relative luciferase activity of callus carrying the Xo2 reporter system 1-6 days after inoculation of the PXO61. Error bars indicate SD (n=5).

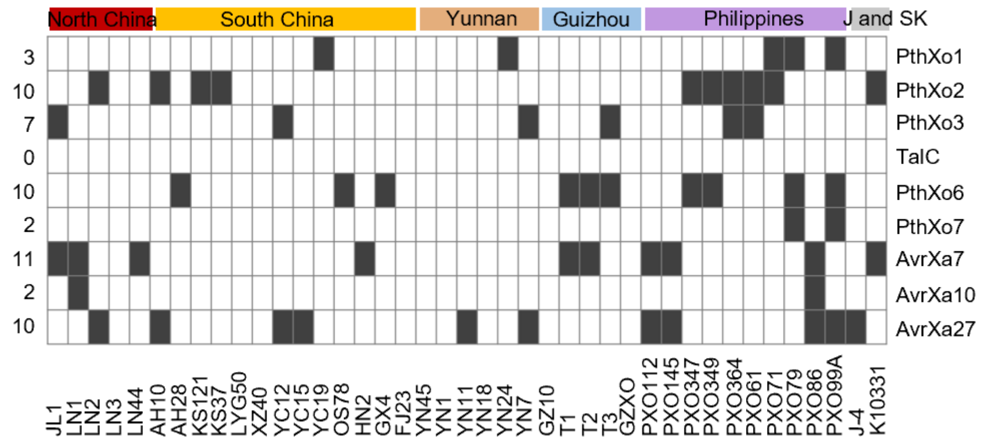


**Supplementary Fig S10. Distribution of TALEs targeting susceptible genes and E genes across 41 *Xoo* strains in this study.** Horizontal axis shows *Xoo* strains. Vertical axis shows TALE genes. Black shading indicates strains containing the corresponding TALE. Colored bars indicate countries where the strains were isolated.

a

| TALE | RVD sequence |
|---|---|
| TalS1 | NI-H*-NI-NN-NN-NN-NN-NN-HD-NI-NS-HG-HD-NI-N*-NS-NI-NI-HD-HD-N*-NS-N* |
| TalS2 | NI-HG-NI-NG-HG-HD-NS-NG-HD-NN-NG-HG-NG-HD-HG-HD-HD-NI-NN-NG |
| TalS3 | NI-N*-NI-HG-NI-NI-NS-HD-NN-HD-NS-NG-SS-HD-NI-NI-NN-NI-NN-NI-NG |
| TalS4 | NI-HG-NI-NN-NN-NI-NN-HD-NI-HD-NS-NS-NS-HD-NN-HD-NG-HD-HD-HD-NG-NG |
| TalS5 | NI-N*-NI-NS-NN-NG-NN-NS-N*-NS-NN-NS-N*-HD-HG-HD-NI-HD-HD-NG |

b

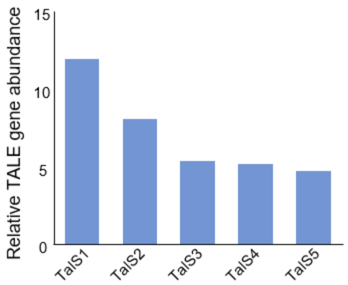


**Supplementary Fig S11. TALE genes and RVD sequences identified by HiFi sequencing. (a)** TALE genes and RVD sequences identified by HiFi sequencing. **(b)** Relative abundance of the top five TALE genes identified by HiFi sequencing, shown as the percentage of a certain TALE gene compared to all TALE genes identified in 30 *Xoo* strains.

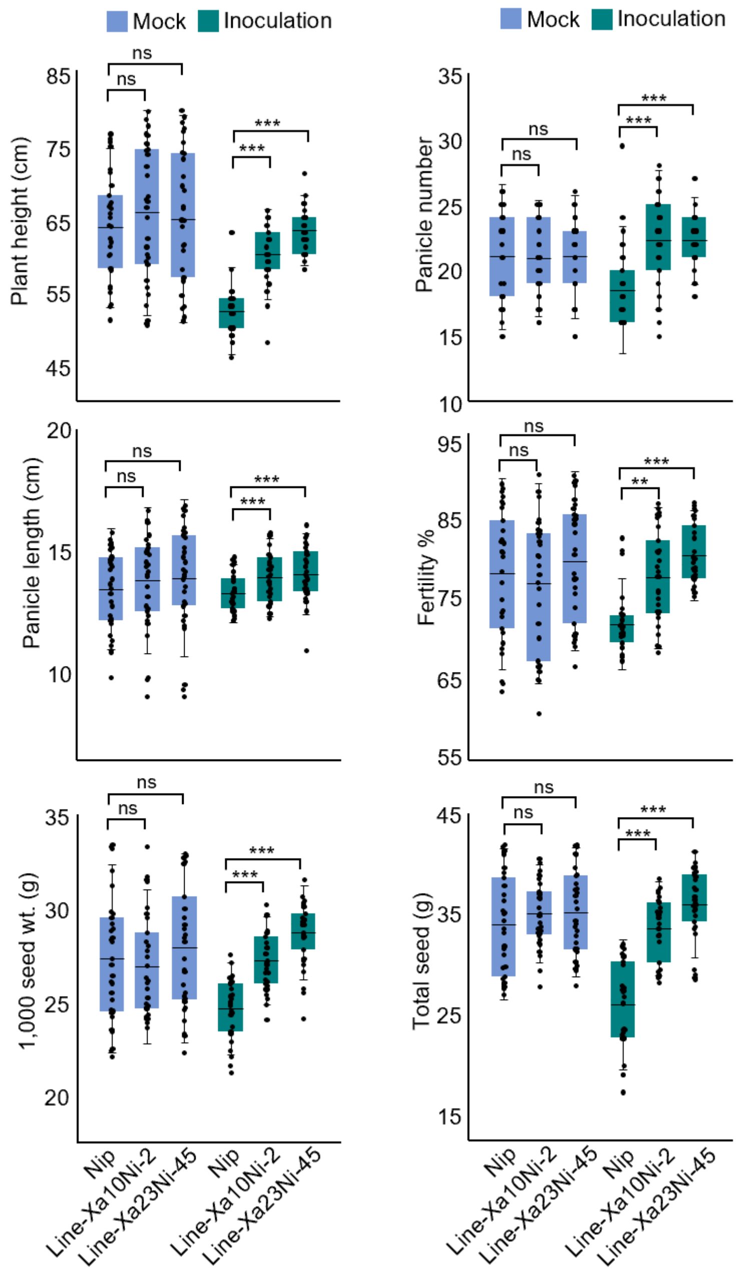


**Supplementary Fig S 12. Agronomic traits of *Xa10Ni-2* and *Xa23Ni-45* $T_4$ lines.** Error bars indicate SD (n=30). *p*-values from *t*-tests at a significance level of 0.05.

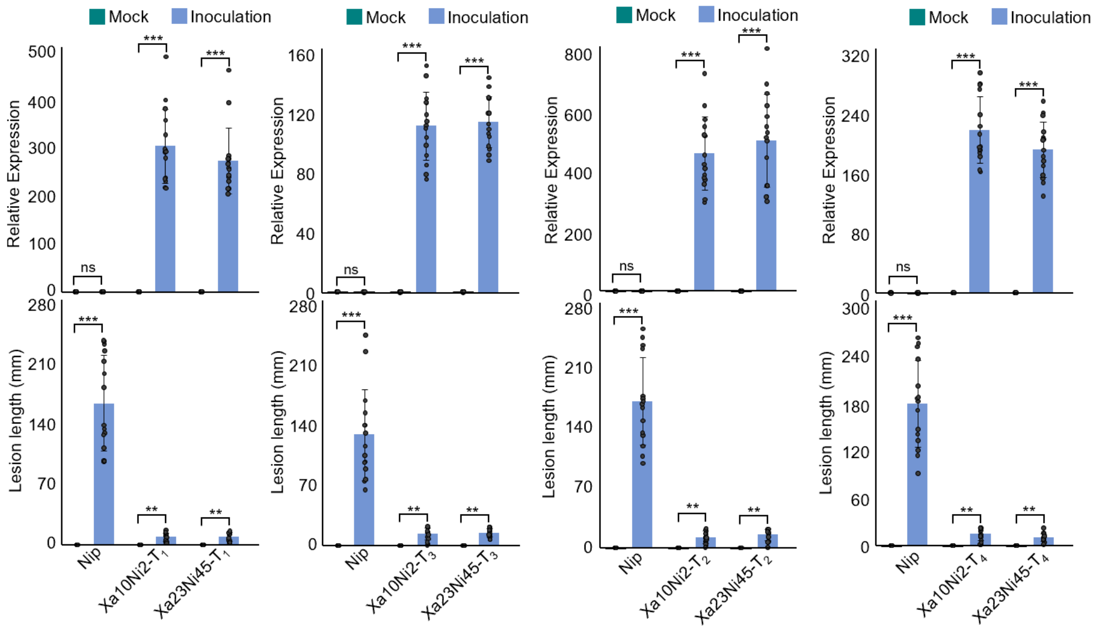


**Supplementary Fig S13. Multi-generation resistance monitoring of knock-in lines.** E gene expression 24 h after inoculation and lesion lengths 14 days after inoculation in *Xa10Ni-2* and *Xa23Ni-45* $T_1$-$T_4$ lines. Error bars indicate SD (n=15). *p*-values from *t*-tests at a significance level of 0.05.

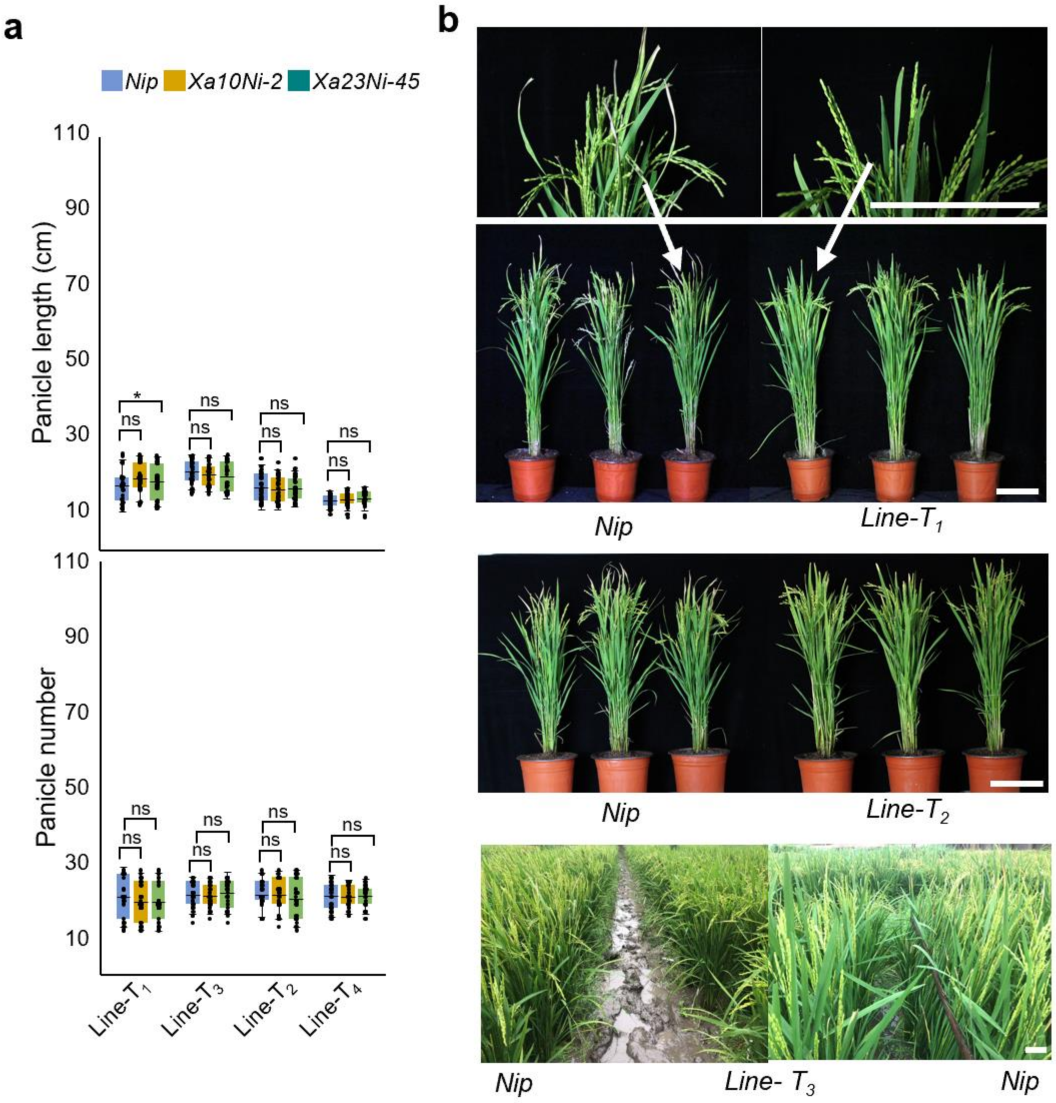


**Supplementary Fig S14. Agronomic traits and phenotype of *Xa10Ni-2* and *Xa23Ni-45* $T_1$-$T_4$ lines.** **(a)** Agronomic traits of *Xa10Ni-2* and *Xa23Ni-45* $T_1$-$T_4$ lines. $T_1$ and $T_3$ generations were grown in Hainan, $T_2$ and $T_4$ in Shanghai, Error bars indicate SD (n=15), *p*-values from *t*-tests at a significance level of 0.05. **(b)** Phenotypes of *Xa10Ni-2* and *Xa23Ni-45* $T_1$-$T_3$ plants. Arrows indicate magnified views of inoculated areas, scale bar, 15cm.

## Supplementary Table

**SupplementaryTable S1. Summary of genome editing results in $T_0$ transgenic rice**

| Target | | | Donor | | $T_0$ plants | | |
|---|---|---|---|---|---|---|---|
| **Locus** | **Region** | **sgRNA** | **Name** | **Length** | **No.** | **Total** | **Knock-in%** |
| Xa10Ni | -138 bp from ATG | sgRNA-1 | Dn1#E1Xo2 | 40 | 8 | 77 | 10.4 |
| | | | Dn2#E1Xa7 | 40 | 12 | 90 | 13.3 |
| | | | Dn3#E1Xa10 | 40 | 9 | 65 | 13.8 |
| | | | Dn4#E2Xa10&Xa7 | 70 | 13 | 92 | 14.1 |
| | | | Dn5#E3Xo2&Xa7&Xo6 | 90 | 13 | 56 | 23.2 |
| | | | Dn6#E4Xo7&Xa10&Xo6&Xa27 | 100 | 16 | 128 | 12.5 |
| | | | Dn7#E4Xo1&Xo2&TalC&Xa7 | 100 | 18 | 127 | 14.2 |
| | | | Dn8#E4Tal10&Tal15&TalS1&TalS2 | 110 | 11 | 96 | 11.5 |
| Xa23Ni | -110 bp from ATG | sgRNA-2 | Dn1#E1Xo2 | 40 | 13 | 106 | 12.3 |
| | | | Dn2#E1Xa7 | 40 | 15 | 85 | 17.6 |
| | | | Dn3#E1Xa10 | 40 | 15 | 144 | 10.4 |
| | | | Dn4#E2Xa10&Xa7 | 70 | 9 | 60 | 15.0 |
| | | | Dn5#E3Xo2&Xa7&Xo6 | 90 | 13 | 75 | 17.3 |
| | | | Dn6#E4Xo7&Xa10&Xo6&Xa27 | 100 | 7 | 133 | 5.3 |
| | | | Dn7#E4Xo1&Xo2&TalC&Xa7 | 100 | 14 | 112 | 12.5 |
| | | | Dn8#E4Tal10&Tal15&TalS1&TalS2 | 110 | 10 | 68 | 14.7 |
| Xa27Ni | -119 bp from ATG | sgRNA-3 | Dn1#E1Xo2 | 40 | 6 | 52 | 11.5 |
| | | | Dn2#E1Xa7 | 40 | 11 | 83 | 13.3 |
| | | | Dn3#E1Xa10 | 40 | 12 | 140 | 8.6 |
| | | | Dn4#E2Xa10&Xa7 | 70 | 9 | 105 | 8.6 |
| | | | Dn5#E3Xo2&Xa7&Xo6 | 90 | 13 | 90 | 14.4 |
| | | | Dn6#E4Xo7&Xa10&Xo6&Xa27 | 100 | 16 | 113 | 14.2 |
| | | | Dn7#E4Xo1&Xo2&TalC&Xa7 | 100 | 16 | 109 | 14.7 |

**SupplementaryTable S2. The *Xanthomonas oryzae pv oryzae* strains used in this study.**

| Location of East Asia | Strain | Geolocation | Accession number |
|---|---|---|---|
| North China | JL1 | China-Jilin | SAMN40559719 |
| | LN1 | China-Liaoning | SAMN40559750 |
| | LN2 | China-Liaoning | SAMN40559775 |
| | LN3 | China-Liaoning | SAMN40559776 |
| | LN44 | China-Liaoning | SAMN40559777 |
| Yangtze Valley of China | AH10 | China-Anhui | SAMN40559779 |
| | AH28 | China-Anhui | SAMN40559780 |
| | KS37 | China-Jiangsu | SAMN40560098 |
| | LYG50 | China-Jiangsu | SAMN40560123 |
| | XZ40 | China-Jiangsu | SAMN40560154 |
| | YC12 | China-Jiangsu | SAMN40560157 |
| | YC15 | China-Jiangsu | SAMN40560159 |
| | YC19 | China-Jiangsu | SAMN40560160 |
| | KS121 | China-Jiangsu | SAMN40559781 |
| | OS78 | China-Zhejiang | SAMN40560376 |
| | HN2 | China-Hunan | SAMN40560391 |
| South China | FJ23 | China-Fujian | SAMN40560406 |
| | GX4 | China-Guangxi | SAMN40560393 |
| | GZ10 | China-Guizhou | SAMN40560514 |
| | GZXO | China-Guizhou | SAMN40560522 |
| | T1 | China-Guizhou | SAMN40560516 |
| | T2 | China-Guizhou | SAMN40560518 |
| | T3 | China-Guizhou | SAMN40560520 |
| | YN1 | China-Yunnan | SAMN40560500 |
| | YN11 | China-Yunnan | SAMN40560502 |
| | YN18 | China-Yunnan | SAMN40560503 |
| | YN45 | China-Yunnan | SAMN40560498 |
| | YN7 | China-Yunnan | SAMN40560504 |
| | YN24 | China-Yunnan | SAMN06027758 |
| Philippines | PXO112 | Philippines | SAMN40560524 |
| | PXO347 | Philippines | SAMN40560526 |
| | PXO349 | Philippines | SAMN40560528 |
| | PXO61 | Philippines | SAMN06695275 |
| | PXO71 | Philippines | SAMN04306145 |
| | PXO79 | Philippines | SAMN05953163 |
| | PXO145 | Philippines | SAMN04306146 |
| | PXO86 | Philippines | SAMN02616002 |
| | PXO99A | Philippines | SAMN02603061 |
| | PXO364 | Philippines | SAMN10261819 |
| Japan | J4 | Japan | SAMN40560530 |

| | | | |
|---|---|---|---|
| South Korea | KACC10331 | South Korea | SAMN02603680 |

**Supplementary Table S3. RVD sequences of conserved TALEs in 84 strains of *Xoo***

| TALE | RVD sequence |
|---|---|
| Tal10-45 | NI-NG-NN-NG-NK-NG-NI-NN-NI-NN-NI-NN-NS-NG-NS-NN-NI-N*-NS-NG |
| Tal11-47 | NI-NN-NI-HG-HG-HD-NG-HD-HG-HD-HD-HD-NG |
| Tal12-47 | NI-NN-NN-NI-NI-NI-HD-NS-HG-NN-NN-NN-NI-NI-HG-HD |
| Tal13-37 | NI-NS-HD-NG-NS-NN-HD-N*-NN-NN-NI-NG-HD-NG-HD-HD-HD-NG |
| Tal15-45 | NN-HD-NS-NG-HD-NN-N*-NI-HD-NS-HD-NN-HD-NN-HD-NN-NN-NN-NN-NN-NN-NN-HD-NG |
| Tal4-27 | NI-HD-NN-NS-NN-NG-HD-NG-HD-NG-NN-NG-HD-NN-HD-NI-NG-HD-HD-NN-HD-NN |
| Tal16-23 | NN-HD-NV-HD-NI-NG-NI-NN-NS-HD-HD-NI-NG-NI-NG-NI |
| Tal17-27 | NS-NG-NS-HD-NI-NG-NN-NG-HD-NI-NN-N*-NI-NN-HD-NG-NI-NN-N*-HD-NN-NG |

**Supplementary Table S4. sgRNAs and primers used in this study**

| Name | Sequence |
|---|---|
| sgRNA-1 | CTAAAGGCGCTGAAAGAAAA |
| sgRNA-2 | ATGTTAGTGAGGCGGAAGGA |
| sgRNA-3 | TCTTGTGTACTTGTGAATTG |
| Xa10Ni-gtF | CATGCAGCGTCCTGGAGTGG |
| Xa10Ni-gtR | GGACGGCGATGAGGAGAAGC |
| Xa23Ni-gtF | TCTCACAGCTAACCCGAACA |
| Xa23Ni-gtR | GGAGGAGAAAGCGGCAGAGG |
| Xa27Ni-gtF | GATTCGTCACTGCCCATCAA |
| Xa27Ni-gtR | AGGAGGTAGTGGTGCATCGC |
| Xa10Ni-qF | GCCATCTTCGCCTTCTTCTTC |
| Xa10Ni-qR | ATGAGCGCGAGAGTGACATA |
| Xa23Ni-qF | ATGTCTCTCGGTCATGCTGT |
| Xa23Ni-qR | GCCCTGAAGGCTTAAACAGG |
| Xa27Ni-qF | CTCCTACTAGCCAACCAGCA |
| Xa27Ni-qR | GAGCAGGTGCATGAGGATGA |
| Actin1-qF | CTGCGGGTATCCATGAGACT |
| Actin1-qR | AAGGCAGTGATCTCCTTGCT |
| SPS1-sF | ATCCTGCTCTTGTTGAGCCT |
| SPS1-sR | CAAGCTGGCACTCCTGATTC |
| Cas9-sF | GCATCCTGCAGACAGTGAAG |
| Cas9-sR | TATTCTCGGCCTGCTCTCTG |
| SPS1-F | CGTGTGCGTATTCATGGTGT |
| SPS1-R | CCACGAGTAGAGCTGGATGT |
| Cas9-F | GCATCCTGCAGACAGTGAAG |
| Cas9-R | GCTCTTTGATGCCCTCTTCG |
| Hyg-F | ATATGCGCGATTGCTGATCC |
| Hyg-R | TCCGTCAGGACATTGTTGGA |
| EBE-F | ATGTGTAGAGAGATAGACATGC |
| EBE-R | TAGCGAGTGGTGCTTGG |

**Supplementary Table S5. The location and accession numbers of strains used in the population genetic analysis**

| Country/Region | Strain | Geolocation | Accession number |
|---|---|---|---|
| Philippines | PXO142 | Philippines | SAMN09791863 |
| | PXO211 | Philippines | SAMN04306147 |
| | PXO236 | Philippines | SAMN04306148 |
| | PXO282 | Philippines | SAMN04306149 |
| | PXO404 | Philippines | SAMN10261820 |
| | PXO421 | Philippines | SAMN10261821 |
| | PXO513 | Philippines | SAMN10261822 |
| | PXO524 | Philippines | SAMN04306150 |
| | PXO563 | Philippines | SAMN04306151 |
| | PXO602 | Philippines | SAMN04306152 |
| | PXO83 | Philippines | SAMN04161335 |
| South Korea | K1 | South Korea | SAMN14167900 |
| | J18 | South Korea | SAMN16744833 |
| | JW11089 | South Korea | SAMN10261813 |
| | K2 | South Korea | SAMN14361487 |
| | K3 | South Korea | SAMN14362543 |
| | K3a | South Korea | SAMN14362545 |
| Japan | JP01 | Japan | SAMN05953100 |
| | MAFF 311018 | Japan | SAMD00061078 |
| | T7133 | Japan | SAMN18324990 |
| India | BXO1 | India | SAMN03252489 |
| | BXO512 | India | SAMN03252503 |
| | ICMP3125 | India | SAMN09791860 |
| | ITCCBB0002 | India | SAMN13318517 |
| | IX280 | India | SAMN05717683 |
| | IXO1088 | India | SAMN03252586 |
| | IXO704 | India | SAMN03252585 |
| | KKXoo | India | SAMN28690421 |
| Thailand | CR1-5 | Thailand | SAMN06256029 |
| | SK2-3 | Thailand | SAMN06255990 |
| Mali | CFBP1949 | Mali | SAMN10254783 |
| | CFBP1951 | Mali | SAMN10254784 |
| | CFBP1952 | Mali | SAMN10254785 |
| | CFBP7337 | Mali | SAMN10254793 |
| | MAI1 | Mali | SAMN08225246 |
| | MAI106 | Mali | SAMN05757032 |
| | MAI129 | Mali | SAMN05757033 |
| | MAI134 | Mali | SAMN05757034 |
| | MAI145 | Mali | SAMN05757035 |

| | | | |
|---|---|---|---|
| | MAI68 | Mali | SAMN05757028 |
| | MAI73 | Mali | SAMN05757029 |
| | MAI95 | Mali | SAMN05757030 |
| | MAI99 | Mali | SAMN05757031 |
| Niger | CFBP7323 | Niger | SAMN10254790 |
| | CFBP7324 | Niger | SAMN10254791 |
| | CFBP7325 | Niger | SAMN10254792 |
| | CFBP7340 | Niger | SAMN10254794 |
| | CIX298 | Niger | SAMN10960780 |
| Tanzania | Dak16 | Tanzania | SAMN10254796 |
| | T19 | Tanzania | SAMN10254797 |
| Uganda | Ug11 | Uganda | SAMN10254798 |
| Cameroon | AXO1947 | Cameroon | SAMN04287888 |
| | CFBP1948 | Cameroon | SAMN10254782 |
| Burkina Faso | BAI3 | Burkina Faso | SAMN08225245 |
| | CIX2374 | Burkina Faso | SAMN10960779 |
| | CFBP7319 | Burkina Faso | SAMN10254786 |
| North China | JL15 | China-Jilin | SAMN05953080 |
| | JL16 | China-Jilin | SAMN05953082 |
| | JL19 | China-Jilin | SAMN05953084 |
| | JL21 | China-Jilin | SAMN05953085 |
| | JL23 | China-Jilin | SAMN05953087 |
| | JL29 | China-Jilin | SAMN05953091 |
| | JL3 | China-Jilin | SAMN05953092 |
| | JL31 | China-Jilin | SAMN05953095 |
| | JL4 | China-Jilin | SAMN05953097 |
| | JL6 | China-Jilin | SAMN05953098 |
| | LN8559 | China-Liaoning | SAMN05953121 |
| | DBX0016 | China-Liaoning | SAMN05952996 |
| | LN8547 | China-Liaoning | SAMN05953120 |
| | LNXO02 | China-Liaoning | SAMN05953116 |
| | LNXO01 | China-Liaoning | SAMN05953115 |
| | DBX004 | China-Liaoning | SAMN05953003 |
| | DBX0024 | China-Liaoning | SAMN05953002 |
| | DBX0020 | China-Liaoning | SAMN05953000 |
| | DBX0023 | China-Liaoning | SAMN05953001 |
| | DBX0019 | China-Liaoning | SAMN05952999 |
| | DBX0018 | China-Liaoning | SAMN05952998 |
| | DBX0013 | China-Liaoning | SAMN05952995 |
| | DBX0017 | China-Liaoning | SAMN05952997 |
| | DBX0011 | China-Liaoning | SAMN05952994 |
| Yangtze Valley of China | AH1 | China-Anhui | SAMN05952983 |
| | AH11 | China-Anhui | SAMN05952984 |

| | | | |
|---|---|---|---|
| | AH3 | China-Anhui | SAMN05952985 |
| | AH39 | China-Anhui | SAMN05952986 |
| | AH4 | China-Anhui | SAMN05952987 |
| | AH40 | China-Anhui | SAMN05952988 |
| | AH8 | China-Anhui | SAMN05952989 |
| | AH87 | China-Anhui | SAMN05952990 |
| | HeN03-01 | China-Henan | SAMN05953044 |
| | HeN03-04 | China-Henan | SAMN05953047 |
| | HeN03-06 | China-Henan | SAMN05953048 |
| | HeN03-10 | China-Henan | SAMN05953049 |
| | HeN03-11 | China-Henan | SAMN05953050 |
| | HeN03-16 | China-Henan | SAMN05953051 |
| | HEN08 | China-Henan | SAMN05953052 |
| | HEN11 | China-Henan | SAMN03020242 |
| | HEN12 | China-Henan | SAMN05953054 |
| | JS72 | China-Jiangsu | SAMN05953108 |
| | LYG46 | China-Jiangsu | SAMN05953130 |
| | LYG48 | China-Jiangsu | SAMN05953131 |
| | LYG51 | China-Jiangsu | SAMN05953133 |
| | LYG52 | China-Jiangsu | SAMN05953134 |
| | XZ40 | China-Jiangsu | SAMN05953174 |
| | XZ43 | China-Jiangsu | SAMN05953176 |
| | YC26 | China-Jiangsu | SAMN05953180 |
| | YC3 | China-Jiangsu | SAMN05953181 |
| | HuN39 | China-Hunan | SAMN05953074 |
| | HUN22 | China-Hunan | SAMN05953068 |
| | HUN20 | China-Hunan | SAMN05953067 |
| | HN7 | China-Hunan | SAMN05953059 |
| | HuN38 | China-Hunan | SAMN05953071 |
| | ScHy-b | China-Sichuan | SAMN05953170 |
| | OS166 | China-Sichuan | SAMN05953138 |
| | ZJ25 | China-Zhejiang | SAMN05953228 |
| | ZJ16 | China-Zhejiang | SAMN05953227 |
| | OS181 | China-Zhejiang | SAMN05953140 |
| | ZPY1 | China-Zhejiang | SAMN05953232 |
| | ZJ28 | China-Zhejiang | SAMN05953231 |
| | ZJ26 | China-Zhejiang | SAMN05953229 |
| South China | YN24 | China-Yunnan | SAMN03020249 |
| | WHRI 5234 | China-Yunnan | SAMN08729510 |
| | YN8 | China-Yunnan | SAMN03020248 |
| | YN6 | China-Yunnan | SAMN05953203 |
| | YN40 | China-Yunnan | SAMN05953221 |
| | YN20 | China-Yunnan | SAMN05953216 |

| | | | |
|---|---|---|---|
| | YN19 | China-Yunnan | SAMN05953215 |
| | YN17 | China-Yunnan | SAMN05953214 |
| | YN11 | China-Yunnan | SAMN05953209 |
| | YN07 | China-Yunnan | SAMN05953204 |
| | YN03-23 | China-Yunnan | SAMN05953199 |
| | FJ22 | China-Fujian | SAMN05953008 |
| | FJ24 | China-Fujian | SAMN05953009 |
| | FJ21 | China-Fujian | SAMN05953007 |
| | FJ17 | China-Fujian | SAMN05953006 |
| | FJ16 | China-Fujian | SAMN05953005 |
| | OS34 | China-Fujian | SAMN05953152 |
| | XF89b | China-Taiwan | SAMN03729481 |
| | XM9 | China-Taiwan | SAMN06480557 |
| | OS54 | China-Guangxi | SAMN05953156 |
| | GX6 | China-Guangxi | SAMN05953039 |
| | GX3 | China-Guangxi | SAMN05953033 |
| | GX2 | China-Guangxi | SAMN05953031 |
| | GX09 | China-Guangxi | SAMN05953029 |
| | GX07 | China-Guangxi | SAMN05953028 |
| | GX56 | China-Guangxi | SAMN05953038 |
| | GX5 | China-Guangxi | SAMN05953037 |
| | GX49 | China-Guangxi | SAMN05953036 |
| | GX35 | China-Guangxi | SAMN05953034 |
| | GD414 | China-Guangdong | SAMN03020241 |
| | GD416 | China-Guangdong | SAMN05953026 |
| | GD413 | China-Guangdong | SAMN05953024 |
| | GD407 | China-Guangdong | SAMN05953019 |
| | GD403 | China-Guangdong | SAMN05953016 |
| | GD402 | China-Guangdong | SAMN05953015 |
| | GD401 | China-Guangdong | SAMN05953014 |
| | GD32 | China-Guangdong | SAMN05953011 |
| | GD417 | China-Guangdong | SAMN05953027 |
| | GD415 | China-Guangdong | SAMN05953025 |
| | GD412 | China-Guangdong | SAMN05953023 |

**Supplementary Table S6. The resistance/susceptible phenotypes of lines E4-11 and E4-14 to the 41 strains**

| Strain | Presence/absence of TALEs can be trapped by E4-11 (Xo7, Xa10, Xo6, Xa27) | Presence/absence of TALEs can be trapped by E4-14 (Xo1, Xo2, Xa7, Xo3, TalC) | Resistance/susceptible phenotypes of lines E4-11 or E4-14 | |
|---|---|---|---|---|
| | | | E4-11 | E4-14 |
| AH28 | Xo6 | absence | R | S |
| J-4 | Xa27 | absence | R | S |
| OS78 | Xo6 | absence | R | S |
| YC15 | Xa27 | absence | R | S |
| GX4 | Xo6 | absence | R | S |
| YN11 | Xa27 | absence | R | S |
| YC19 | absence | Xo1 | S | R |
| HN-2 | absence | Xa7 | S | R |
| JL1 | absence | Xo3, Xa7 | S | R |
| YN24 | absence | Xo1 | S | R |
| KS37 | absence | Xo2 | S | R |
| KS121 | absence | Xo2 | S | R |
| LN44 | absence | Xa7 | S | R |
| PXO61 | absence | Xo2, Xo3 | S | R |
| PXO71 | absence | Xo1, Xo2 | S | R |
| K10331 | absence | Xo2, Xa7 | S | R |
| PXO364 | absence | Xo2, Xo3 | S | R |
| LN3 | absence | absence | S | S |
| XZ40 | absence | absence | S | S |
| YN18 | absence | absence | S | S |
| YN1 | absence | absence | S | S |
| YN45 | absence | absence | S | S |
| GZXO | absence | absence | S | S |
| FJ23 | absence | absence | S | S |
| LYG50 | absence | absence | S | S |
| GZ10 | absence | absence | S | S |
| PXO112 | Xa27 | Xa7 | R | R |
| PXO145 | Xa27 | Xa7 | R | R |
| PXO347 | Xo6 | Xo2 | R | R |
| PXO349 | Xo6 | Xo2 | R | R |
| PXO79 | Xo6 | Xo7 | R | R |
| PXO86 | Xa10 Xa27 | Xa27 | R | R |
| PXO99A | Xo6 Xo7 Xa27 | Xo1 | R | R |
| LN1 | Xa10 | Xa7 | R | R |

| | | | | |
|---|---|---|---|---|
| LN2 | Xa27 | Xo2 | R | R |
| AH10 | Xa27 | Xo2 | R | R |
| YC12 | Xa27 | Xo3 | R | R |
| YN7 | Xa27 | Xo3 | R | R |
| T1 | Xo6 | Xa7 | R | R |
| T2 | Xo6 | Xa7 | R | R |
| T3 | Xo6 | Xo3 | R | R |

**Supplementary Table S7. The copy number of EBE, Cas9, and Hyg of lines *Xa10Ni-2* and *Xa23Ni-45***

| Lines | Sample | EBE | Cas9 | Hyg | Sample | EBE | Cas9 | Hyg |
|---|---|---|---|---|---|---|---|---|
| T0 | T0-Xa10Ni-2 | 2 | 1 | 1 | T0-Xa23Ni-29 | 5 | 1 | 1 |
| | T0-Xa10Ni-26 | 2 | 1 | 1 | T0-Xa23Ni-36 | 7 | 1 | 1 |
| | T0-Xa10Ni-27 | 13 | 1 | 1 | T0-Xa23Ni-45 | 2 | 1 | 2 |
| | T0-Xa10Ni-45 | 3 | 1 | 1 | T0-Xa23Ni-68 | 2 | 2 | 2 |
| | T0-Xa10Ni-81 | 4 | 1 | 2 | T0-Xa23Ni-90 | 16 | 5 | 6 |
| T1 | T1-Xa10Ni-2-1 | 2 | 1 | 1 | T1-Xa23Ni-45-1 | 2 | 1 | 2 |
| | T1-Xa10Ni-2-2 | 2 | 1 | - | T1-Xa23Ni-45-2 | 2 | 1 | 1 |
| | T1-Xa10Ni-2-3 | 1 | 1 | - | T1-Xa23Ni-45-3 | 2 | - | 1 |
| | T1-Xa10Ni-2-4 | 2 | 1 | 1 | T1-Xa23Ni-45-4 | 2 | 1 | 1 |
| | T1-Xa10Ni-2-5 | 2 | - | 2 | T1-Xa23Ni-45-5 | 2 | 1 | 2 |
| T2 | T2-Xa10Ni-2-1 | 1 | - | - | T2-Xa23Ni-45-1 | 2 | 1 | - |
| | T2-Xa10Ni-2-2 | 1 | - | - | T2-Xa23Ni-45-2 | 2 | - | 1 |
| | T2-Xa10Ni-2-3 | 1 | - | - | T2-Xa23Ni-45-3 | 2 | 1 | 1 |
| | T2-Xa10Ni-2-4 | 1 | - | - | T2-Xa23Ni-45-4 | 2 | 1 | 1 |
| | T2-Xa10Ni-2-5 | 1 | - | - | T2-Xa23Ni-45-5 | 2 | 1 | 1 |
| T3 | T3-Xa10Ni-2-1 | 1 | - | - | T3-Xa23Ni-45-1 | 2 | - | - |
| | T3-Xa10Ni-2-2 | 1 | - | - | T3-Xa23Ni-45-2 | 2 | - | - |
| | T3-Xa10Ni-2-3 | 1 | - | - | T3-Xa23Ni-45-3 | 2 | - | - |
| | T3-Xa10Ni-2-4 | 1 | - | - | T3-Xa23Ni-45-4 | 2 | - | - |
| | T3-Xa10Ni-2-5 | 1 | - | - | T3-Xa23Ni-45-5 | 2 | - | - |
| T4 | T4-Xa10Ni-2-1 | 1 | - | - | T4-Xa23Ni-45-1 | 2 | - | - |
| | T4-Xa10Ni-2-2 | 1 | - | - | T4-Xa23Ni-45-2 | 2 | - | - |
| | T4-Xa10Ni-2-3 | 1 | - | - | T4-Xa23Ni-45-3 | 2 | - | - |
| | T4-Xa10Ni-2-4 | 1 | - | - | T4-Xa23Ni-45-4 | 2 | - | - |
| | T4-Xa10Ni-2-5 | 1 | - | - | T4-Xa23Ni-45-5 | 2 | - | - |

**Supplementary Table S8 The off-target estimation of lines *Xa10Ni-2* and *Xa23Ni-45***

| Sort | Chrom | Position | Sequence | Off-score | Gene | Region | Reads number | Variation type | Off-target |
|---|---|---|---|---|---|---|---|---|---|
| Xa10-2-1 | chr01 | 40678214 | GCAAAGGTGCCAAAAGAAAAGGG | 0.426 | | intergenic | 7176 | WT | No |
| Xa10-2-2 | chr02 | 21592640 | CCAAAGACGATGAGAGAAAAGGG | 0.347 | | intergenic | 5545 | WT | No |
| Xa10-2-3 | chr02 | 16601068 | CCAAATGGGCTAAAAGAAAAGGG | 0.343 | | intergenic | 4319 | WT | No |
| Xa10-2-4 | chr11 | 11838349 | TTGGAAGAGCTGAAAGAAAAGGG | 0.248 | | intergenic | 6615 | WT | No |
| Xa10-2-5 | chr03 | 36038364 | CAAAAGGTGCTGAAAGAAGGGGG | 0.212 | | intergenic | 7520 | WT | No |
| Xa10-2-6 | chr09 | 19763638 | CTGAATGCACAGAAAGAAAAGGG | 0.196 | Os09g0509350 | CDS | 7353 | WT | No |
| Xa10-2-7 | chr10 | 9040542 | AGAAAGGGGAGGAAAGAAAAGGG | 0.187 | | intergenic | 6882 | WT | No |
| Xa10-2-8 | chr02 | 23609221 | CAAAAGGGAGTGAAAGAAAAGGG | 0.136 | | intergenic | 4106 | WT | No |
| Xa10-2-9 | chr09 | 8910437 | ATATAGGCACTGAAAGAAAAGAG | 0.106 | | intergenic | 7252 | WT | No |
| Xa10-2-10 | chr02 | 28151228 | CTACAGGCGGTAAAAGAAAAGAG | 0.033 | | intergenic | 7180 | WT | No |
| Xa10-2-11 | chr01 | 3483866 | CTCATGACGCTGAAAGAAAAGGA | 0.011 | Os01g0168100 | intron | 3574 | WT | No |
| Xa10-2-12 | chr10 | 7584514 | CTAATGGCGCTAAAAGGAAGGGA | 0.003 | | intergenic | 7216 | WT | No |
| Xa10-2-13 | chr02 | 9448637 | CAAAAGGAGATGAAACAAAAGGG | 0 | Os02g0265200 | 3_prime_UTR | 7289 | WT | No |
| Xa10-2-14 | chr02 | 663677 | CCAAAGACGATGAAACAAAAGGG | 0 | | intergenic | 6899 | WT | No |
| Xa10-2-15 | chr09 | 445028 | CTAAAGGCGCTCAAACATCAGGT | 0 | Os09g0103600 | 3_prime_UTR | 7021 | WT | No |
| Xa23-45-1 | chr08 | 20221751 | GTGGTGGGGAAGCGGAAGGAAGG | 0.327 | | intergenic | 5113 | WT | No |
| Xa23-45-2 | chr02 | 2063988 | AAGATAGTAAGGCGAAAGGAAGG | 0.244 | EPIOSAG00000008321 | exon | 2829 | WT | No |
| Xa23-45-3 | chr07 | 26015393 | AACTTAGTAAGACGGAAGGAAGG | 0.195 | Os07g0627700 | CDS | 7079 | WT | No |
| Xa23-45-4 | chr08 | 14355471 | TTGTTAGTAAGGAGGAAGAAAGG | 0.177 | | intergenic | 7011 | WT | No |
| Xa23-45-5 | chr04 | 35359786 | TTCTTCGTGAAACGGAAGGAAGG | 0.163 | Os04g0691300 | intron | 7372 | WT | No |
| Xa23-45-6 | chr07 | 26187803 | ATGTTAGAAAGGTGGAACGGAGG | 0.131 | EPIOSAG00000029186 | exon | 7468 | WT | No |
| Xa23-45-7 | chr03 | 16875324 | TTGTCACTGAGCCTGAAGGAAGG | 0.097 | | intergenic | 7567 | WT | No |
| Xa23-45-8 | chr02 | 1087629 | ATGTTAGGGAGGAAGAATTAAGG | 0.094 | Os02g0120900 | intron | 6832 | WT | No |
| Xa23-45-9 | chr08 | 10009715 | ATGTGAGAGAGGCGGACGAAAGG | 0.065 | | intergenic | 4113 | WT | No |
| Xa23-45-10 | chr05 | 23101988 | ATGGTGGTGAGGTGGGAGGGAGG | 0.046 | Os05g0470700 | 5_prime_UTR | 5330 | WT | No |
| Xa23-45-11 | chr02 | 5168054 | GTGATGGCGAGGCGGAAGGAACG | 0.036 | | intergenic | 7047 | WT | No |

| | | | | | | | | | |
|---|---|---|---|---|---|---|---|---|---|
| Xa23-45-12 | chr01 | 34233484 | TTGTTGGGTAGGCGGAAGGAAGA | 0.019 | | intergenic | 7224 | WT | No |
| Xa23-45-13 | chr06 | 29343136 | ATGGTGGTGCGGCGGCAGGAAGG | 0 | Os06g0698748 | CDS | 7058 | WT | No |
| Xa23-45-14 | chr04 | 19000728 | ATGTTCGTGAGGCCAAAGGAAAT | 0 | | intergenic | 7221 | WT | No |
| Xa23-45-15 | chr05 | 28916990 | ATGCTATTGAGGCTGAAGGAATT | 0 | Os05g0581100 | 3_prime_UTR | 6497 | WT | No |

## Supplementary Reference


1. Zhai, Zhiyang et al. Isolation of protoplasts from tissues of 14-day-old seedlings of *Arabidopsis thaliana*. *J. Vis. Exp*. **30**, e1149 (2009).
2. Lu, Yuming et al. Targeted, efficient sequence insertion and replacement in rice. *Nat Biotechnol* **38**, 1402-1407 (2020).
3. Cheng, Haoyu et al. Haplotype-resolved de novo assembly using phased assembly graphs with hifiasm. *Nat Methods* **18**, 170-175 (2021).
4. Grau, J. et al. AnnoTALE: bioinformatics tools for identification, annotation, and nomenclature of TALEs from *Xanthomonas* genomic sequences. *Sci Rep* **6**, 21077 (2016).
5. Ivica Letunic et al. Interactive Tree Of Life (iTOL) v5: an online tool for phylogenetic tree display and annotation. *Nucleic Acids Res* **49**, 293–296. (2021)
6. Stamatakis, Alexandros. RAxML version 8: a tool for phylogenetic analysis and post analysis of large phylogenies. *Bioinformatics* **30**, 1312–1313. (2014)
7. Yang, Litao et al. Estimating the copy number of transgenes in transformed rice by real-time quantitative PCR. *Plant Cell Rep*. **23**, 759–763. (2005)
8. Xie, Xianrong et al. CRISPR-GE: A Convenient Software Toolkit for CRISPR-Based Genome Editing. *Mol Plant*.;**10**,1246-1249. (2017)
9. Sun, Tingting et al. “Hi-TOM 2.0: an improved platform for high-throughput mutation detection. *Sci China Life Sci.* s11427-024-2555-x. Epub ahead of print. (2024)
